# Anaerobic and Microaerobic Pretreatment for Improving Methane Production From Paper Waste in Anaerobic Digestion

**DOI:** 10.3389/fmicb.2021.688290

**Published:** 2021-07-06

**Authors:** Chao Song, Wanwu Li, Fanfan Cai, Guangqing Liu, Chang Chen

**Affiliations:** ^1^College of Chemical Engineering, Beijing University of Chemical Technology, Beijing, China; ^2^TEDA Institute of Biological Sciences and Biotechnology, Nankai University, Tianjin, China

**Keywords:** paper waste, anaerobic pretreatment, microaerobic pretreatment, microbial community analysis, anaerobic digestion

## Abstract

Having been generated with a tremendous amount annually, paper waste (PW) represents a large proportion in municipal solid waste (MSW) and also a potential source of renewable energy production through the application of anaerobic digestion (AD). However, the recalcitrant lignocellulosic structure poses obstacles to efficient utilization in this way. Recently, anaerobic and microaerobic pretreatment have attracted attention as approaches to overcome the obstacles of biogas production. This study was set out to present a systematic comparison and assessment of anaerobic and microaerobic pretreatment of PW with different oxygen loadings by five microbial agents: composting inoculum (CI), straw-decomposing inoculum (SI), cow manure (CM), sheep manure (SM), and digestate effluent (DE). The hints of microbial community evolution during the pretreatment and AD were tracked by 16S rRNA high-throughput sequencing. The results demonstrated that PW pretreated by DE with an oxygen loading of 15 ml/gVS showed the highest cumulative methane yield (CMY) of 343.2 ml/gVS, with a BD of 79.3%. In addition to DE, SI and SM were also regarded as outstanding microbial agents for pretreatment because of the acceleration of methane production at the early stage of AD. The microbial community analysis showed that *Clostridium sensu stricto 1* and *Clostridium sensu stricto 10* possessed high relative abundance after anaerobic pretreatment by SI, while *Bacteroides* and *Macellibacteroides* were enriched after microaerobic pretreatment by SM, which were all contributable to the cellulose degradation. Besides, aerobic *Bacillus* in SI and *Acinetobacter* in SM and DE probably promoted lignin degradation only under microaerobic conditions. During AD, *VadinBC27*, *Ruminococcaceae Incertae Sedis*, *Clostridium sensu stricto 1*, *Fastidiosipila*, and *Caldicoprobacter* were the crucial bacteria that facilitated the biodegradation of PW. By comparing the groups with same microbial agent, it could be found that changing the oxygen loading might result in the alternation between hydrogenotrophic and acetoclastic methanogens, which possibly affected the methanogenesis stage. This study not only devised a promising tactic for making full use of PW but also provided a greater understanding of the evolution of microbial community in the pretreatment and AD processes, targeting the efficient utilization of lignocellulosic biomass in full-scale applications.

## Introduction

In modern society, paper materials have become indispensable consumer products with a wide variety of civil and industrial uses (Bajpai, 2014). With the unprecedented economic growth and social improvement in the past 20 years, China has become one of the largest paper-producing and paper-consuming countries (NationMaster, 2021), resulting in tremendous production of paper waste (PW) annually. In 2016, the production of PW in China was over 18 million tons, accounting for 9.15% of total municipal solid waste (MSW) (Zhu et al., 2020). At present, the pathway for the environmentally friendly utilization of PW focuses on the recycling and reproduction for new paper products. However, only for limited cycles can the PW be reused due to the decreased fiber strength, whiteness, and quality in the reproduction (Li et al., 2020). Besides, the PW tainted with glue, paint, food wastes, and other residues cannot be recycled, much of which is simply discarded as MSW (Ma et al., 2020; Zhao et al., 2020). Although other treatment methods, such as incineration, landfilling, and composting, have been gradually developed, the environmental impacts and low energy recovery place a heavy burden on their broader popularization (Manfredi et al., 2011; Sanscartier et al., 2012). Therefore, it is of high significance to develop alternative approaches to make the utmost utilization of PW.

For decades, anaerobic digestion (AD) has been emerging as an efficient and viable solution for biowastes to alleviate environmental pollution and produce renewable energy (Wang et al., 2021). Originated from wood, grass, and other plants, PW is mainly composed of lignocellulosic matter and can be applied in AD (Li et al., 2020), whereas, similar to other feedstocks, the intrinsic lignocellulosic structure retards the decomposition of organic matter and negatively affects the biodegradation process (Abraham et al., 2020). Therefore, many pretreatment strategies have been proposed to break the ceiling of their digestibility. Microbial pretreatment, as a means of biological pretreatment, has recently aroused great interest. The various microbes inoculated into the biowaste produce functional enzymes and metabolites, causing the destruction of the lignocellulosic structure. As a result, enzymatic hydrolysis and biomethanation efficiency could be enhanced (Zhao et al., 2019; Lee et al., 2020). Compared with physical and chemical-based pretreatment methods, microbial pretreatment possesses advantages of low cost, safety, and non-pollution along with the absence of intensive heating or chemicals (Carrere et al., 2016; Amin et al., 2017). Up to now, diverse fungi, bacteria, and microbial consortia have been introduced for pretreatment to assist in the bioconversion of biowaste. Kainthola et al. reported that the methane yield of rice straw was significantly increased by microbial pretreatment using *Phanerochaete chrysosporium* (Kainthola et al., 2019). It was reported by Ali et al. that the methane yield of sawdust pretreated by bacterial consortium increased by 92.2% compared with the control (Ali et al., 2019). The constructed microbial consortia described by Patel et al. led to a 3.7-fold polyhydroxybutyrate yield improvement during the dark fermentation of pea-shell slurry (Patel et al., 2015). However, there are still drawbacks undermining the feasibility of microbial pretreatment in large-scale applications. The mild biochemical reactions by microbes make the degradation rate to be slower than that of physical and chemical solutions (Den et al., 2018). Besides, the hydraulic retention time of microbial pretreatment is naturally extended due to microbial growth and propagation, especially for fungi (Amin et al., 2017). Such disadvantages will consequently increase the equipment scale, energy consumption, and capital investment to an extent.

Recently, microaerobic pretreatment, that is, supplying a small amount of oxygen during microbial pretreatment, has been given much attention. Previous studies have shown that under the microaerobic condition, the stimulated microbes showed higher hydrolysis and acidogenesis activity (Lim and Wang, 2013). Therefore, the shorter pretreatment period and lower energy costs can be realized (He et al., 2017; Xu et al., 2018; Wang et al., 2020). However, the microbes for pretreatment were not adaptive to every substrate to improve methane production, nor was it under every oxygen content. For instance, Zhen et al. (2020) stated that cumulative methane yield (CMY) of kitchen wastes did not show any improvement after the microaerobic pretreatment. It was reported by Fu et al. that microaerobic pretreatment with excessive oxygen resulted in decreased methane production of corn straw (Fu et al., 2016). During the microbial pretreatment, there existed a complicated microbial relationship created by a variety of microbes with distinct functions, some of which possibly exhibited different response to the oxygen loading and therefore affected the AD process. Zhen et al. stated that after the microaerobic pretreatment, the *Firmicute* and *Bacteroidetes* were the predominant phyla during the AD of rice straw (Zhen et al., 2021). However, Ruan et al. (2019) found that the digestate from the anaerobic sludge digestion possessed higher abundances of *Proteobacteria* and *Chloroflexi*. It can be deduced that the substrate, microbial consortia, and oxygen loadings are crucial factors that contribute to this difference. However, the systematic comparison in terms of these factors was still insufficient due to the various experimental conditions. In this work, five different microbial agents enriched with various microbes, namely, composting inoculum (CI), straw-decomposing inoculum (SI), cow manure (CM), sheep manure (SM), and digestate effluent (DE), were introduced to achieve the following aims:

1. Construct an optimal microbial pretreatment tactic that combines the microbial agent with suitable oxygen loading to attain improved methane production performance of PW.
2. Explore the evolution of the microbial community in anaerobic pretreatment, microaerobic pretreatment, and AD.
3. Propose the functional microbes that synergistically facilitate the biodegradation of lignocellulosic materials during the pretreatment and AD.

## Materials and Methods

### Substrates, Microbial Agents, and Inoculum

The corrugated board (CB) and tissue paper (TP) bought in a supermarket were cut into pieces about 5 mm in length. The CI and SI were liquid and solid microbial consortia commercially designed for composting crop residues. The CM and SM were directly delivered from a farm in Shunyi District, Beijing. DE and inoculum for AD were the activated anaerobic sludge obtained from a biogas plant in Shunyi District, Beijing, which was naturally degassed for 20 days to eliminate the residual biogas. The characteristics of the substrates and microbial agents are shown in Table 1.

**TABLE 1:** Characteristics of substrates and microbial agents.

Parameters	CB	TP	CI	SI	CM	SM	DE
TS (%)^a^	95.54 ± 0.09	97.92 ± 0.02	1.55 ± 0.02	86.39 ± 0.06	28.56 ± 1.10	15.93 ± 0.30	17.03 ± 0.03
VS (%)^a^	93.44 ± 0.08	97.46 ± 0.05	1.20 ± 0.03	22.20 ± 0.60	11.77 ± 0.27	13.89 ± 0.42	9.30 ± 0.04
VS/TS (%)	97.76	99.53	77.43	25.69	41.20	87.16	54.61
pH	NA	NA	6.77 ± 0.04	NA	8.26 ± 0.20	7.83 ± 0.14	8.45 ± 0.09
Cellulose (%)^b^	51.84 ± 0.13	90.92 ± 0.39	NA	NA	9.44 ± 0.81	18.98 ± 0.86	NA
Hemicellulose (%)^b^	8.65 ± 0.71	4.66 ± 0.96	NA	NA	8.94 ± 0.14	27.12 ± 0.73	NA
Lignin (%)^b^	20.71 ± 0.69	1.11 ± 1.46	NA	NA	10.71 ± 0.75	6.63 ± 0.18	NA
C (%)^b^	45.49 ± 0.14	42.90 ± 0.06	NA	8.86 ± 0.45	19.82 ± 0.89	35.83 ± 0.94	28.82 ± 0.13
H (%)^b^	5.96 ± 0.11	5.99 ± 0.08	NA	1.04 ± 0.07	2.46 ± 0.21	5.50 ± 0.19	4.38 ± 0.49
O (%)^b^	43.92 ± 0.65	48.46 ± 0.27	NA	9.84 ± 1.03	20.53 ± 0.29	38.67 ± 0.37	26.95 ± 1.07
N (%)^b^	0.13 ± 0.02	0.15 ± 0.03	NA	0.73 ± 0.08	0.93 ± 0.01	2.05 ± 0.41	2.90 ± 0.06
S (%)^b^	0.76 ± 0.02	0.44 ± 0.22	NA	0.25 ± 0.01	0.28 ± 0.06	0.36 ± 0.08	0.93 ± 0.01
C/N	349.90	295.86	NA	12.14	21.31	17.48	9.94
TMY (ml/gVS)	457.54	407.81	NA	356.59	403.36	415.32	443.94

*^*a*^Calculated based on total weight (%). ^*b*^Calculated based on TS (%). NA: not analyzed.*

### Anaerobic and Microaerobic Pretreatment

The pretreatment was conducted in 20 digesters, which were split into four groups. In each group, the five digesters were filled with each 5 g (based on total solids, TS) of corrugated board and tissue paper and then assigned to the five microbial agents, respectively. Five milliliters of CI and 2 g (based on volatile solids, VS) of SI, CM, SM, and DE were added into assigned digesters. A proper amount of deionized water was also filled into all digesters to reach a TS of 15% and then shaken well to homogenize the mixture. The digesters were purged with high-purity nitrogen (99.99%) for 3 min before a specific amount of pure oxygen was injected into the digesters of each group, that is, 0, 5, 15, and 30 ml/gVS, respectively. All digesters were labeled as “microbial agent—oxygen loading” and placed in a 37°C thermostatic incubator, and the concentrations of oxygen and carbon dioxide were measured every 12 h. The experiments were conducted in triplicate.

### Anaerobic Digestion

**FIGURE 1:**
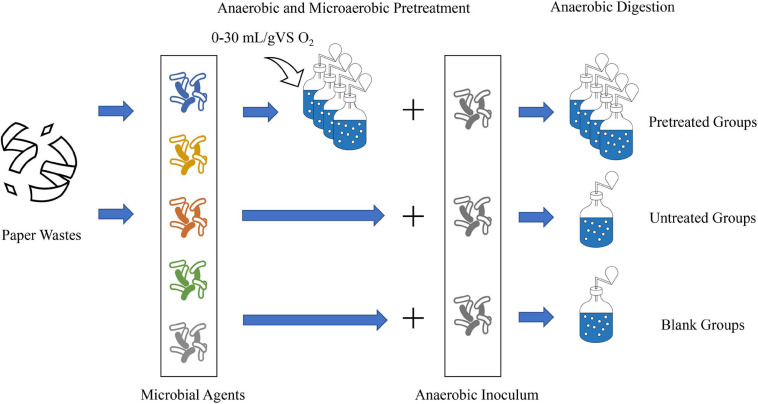
Schematic diagram of the experimental setup.

The preparation of AD started when the oxygen in all digesters was almost exhausted. The substrate-to-inoculum ratio was set to 1:1 (based on VS), and the anaerobic inoculum was added to each digester. Besides, some water was also added into the digester to keep the TS at 15% (organic loading at 47.7 gVS/L). The digesters were purged with pure nitrogen to eliminate other gases, carefully sealed, and placed in a 37°C incubator to start the AD process. Simultaneously, the untreated group (labeled as UN) and blank group for the AD experiment were set up. In the untreated group, PW was directly mixed with inoculum and each microbial agent in five digesters, whose dosages were consistent with what we used for the pretreatment. The blank group was established using five digesters containing inoculum and each microbial agent to calculate the net methane production without the substrates. The untreated and blank groups were also flushed with nitrogen gas and placed in the incubator. Before the biogas pressure test, all digesters were made to shake for 1 min at a low speed. A flowsheet of the experimental setup is shown in Figure 1.

### Analytical Methods

#### Basic Characteristics

The TS and VS for substrates and inoculum could be calculated by Eqs. (1) and (2), based on the standard test methods from APHA (APHA, 2005):

(1) $$\text{TS}=\frac{m_{3}-m_{1}}{m_{2}-m_{1}}\times 100\%$$

(2) $$\text{VS}=\frac{m_{3}-m_{4}}{m_{2}-m_{1}}\times 100\%$$

where *m*_1_ represents the weight of dish (mg), *m*_2_ is equal to the weight of fresh sample and dish (mg), *m*_3_ is the weight of dried residue and dish (mg), and *m*_4_ is assigned to the weight of residue and dish after ignition (mg). A fiber analyzer (ANKOM, New York) was installed to calculate the cellulose, hemicellulose, and lignin contents according to the conventional method (Van Soest et al., 1991; Zhao et al., 2017). The determination of organic elements, including carbon (C), nitrogen (N), hydrogen (H), and sulfur (S), relied on an elemental analyzer (Vario EL cube, Elementar, Germany) (Ning et al., 2018). The mass balance equation supported the calculation of O content, that is, C + H + O + N = 99.5% (based on VS) (Rincón et al., 2012). Since then, it was reasonable to compute the theoretical maximum methane yield (TMY, ml/gVS) of substrates and microbial agents using Buswell’s formula (Li et al., 2018), as shown in Eqs. (3) and (4).

(3) $${\text{C}}_{a}{\text{H}}_{b}{\text{O}}_{c}{\text{N}}_{d}+\left(a-\frac{b}{4}-\frac{c}{2}+\frac{3d}{4}\right){\text{H}}_{2}\text{O}\to \left(\frac{a}{2}+\frac{b}{8}-\frac{c}{4}-\frac{3d}{8}\right){\text{CH}}_{4}+\left(\frac{a}{2}-\frac{b}{8}+\frac{c}{4}+\frac{3d}{8}\right){\text{CO}}_{2}+d{\text{NH}}_{3}$$

(4) $$\text{TMY}=\frac{22.4\times 1000\times \left(\frac{\text{a}}{2}+\frac{\text{b}}{8}-\frac{\text{c}}{4}-\frac{3\text{d}}{8}\right)}{12\text{a}+\text{b}+16\text{c}+14d}$$

#### Production of Methane and Volatile Fatty Acids

The daily biogas yield was calculated following the ideal gas law as shown in Eq. (5) and modified into the standard conditions (0°C, 101 kPa):

(5) $$V_{\text{biogas}}=\frac{P\times V_{\text{headspace}}\times C}{R\times T}$$

where *V*_biogas_ stands for the daily biogas yield (L), *P* refers to the pressure difference (kPa) before and after discharging biogas, *V*_headspace_ is defined as the headspace volume of the digester (L), and *T* is the absolute temperature (K). *C* and *R* are physical constants referring to the molar volume (22.41 L/mol) and universal gas constant (8.314 L kPa/K/mol), respectively.

The biogas compositions and volatile fatty acids (VFAs) contents were measured daily by a gas chromatograph (GC) system (7890B, Agilent, USA), and the detailed instruments were reported elsewhere (Li et al., 2020). The biodegradability (BD) was defined as the ratio of the experimental methane yield (EMY), namely, the highest CMY, divided by the TMY, as shown in Eq. (6):

(6) $$\text{BD}=\frac{\text{EMY}}{\text{TMY}}\times 100\%$$

### Microbial Community Analysis

To identify the functional microbes and evolution of the microbial community during pretreatment and AD, the groups that possessed relatively higher methane yield after AD were selected to conduct the 16S rRNA high-throughput sequencing for the microbial agents, pretreated PW, and digestates after AD. The PCR reaction mixture (30 μl) consisted of 10 ng of extracted DNA, 0.5 μl of Taq DNA polymerase (5 U/μl), 5 μl of deoxyribonucleotide triphosphate (10 mM), and 10 pmol of each primer. 805R (5′-GACTACHVGGGTATCTAATCC-3′) and 341F (5′-15CTACGGGNGGCWGCAG-3′) were considered as the primer pair of amplification for the analysis of total bacteria (Wu et al., 2020). The identification of archaea contained two rounds. 340F (5′-15CCTAYGGGGYGCASCAG-3′) and 1000R (5′-GGCCATGCACYWCYTCTC-3′) were used for the first round of amplification, and 349F (5′-GYGCASCAGKCGMGAAW-3′) and 806R (5′-GGACTACVSGGGTATCTAAT-3′) were used for the second round (Wang et al., 2018).

The Cutadapt software was adopted for the amplicon sequences from the original sequenced fragments. The PEAR program with default settings was adopted for merging the high-quality paired-end reads. The reads abandoned from the raw sequencing data were shorter than 200 bp and contained ambiguous nucleotides, incorrect barcodes, or primers. The UCHIME program was introduced to remove the potential chimera and then cluster the remaining reads into operational taxonomic units (OTUs) with a minimum identity of 97%. The taxonomic assignment of OTUs was supported by the RDP Resource, SILVA rRNA database, and NCBI taxonomy database. The species diversity was estimated by Shannon index, while the species richness was reflected by Chao1 and ACE indexes, which were all calculated using MOTHUR. The relative abundance (RA) was defined as the ratio of the number of sequences affiliated with a taxonomic category to the total number of sequences per sample.

### Statistical Analysis

The results are shown with the average value ± standard deviation and organized by Origin pro 2021 Learning Edition. Circos graphs of microbial community structure were generated by Circos Online^1^.

## Results and Discussion

### Variation in O_2_ and CO_2_ Concentrations During Pretreatment

**FIGURE 2:**
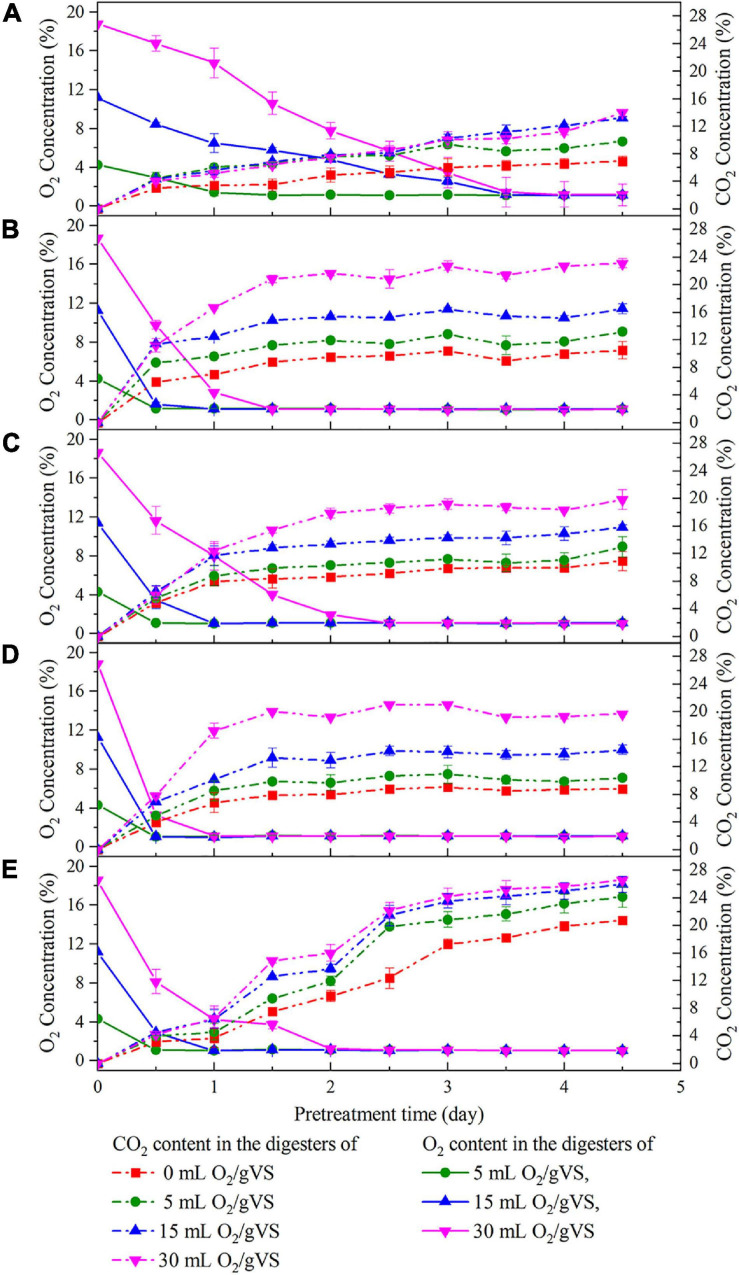
Changes of O_2_ and CO_2_ concentration in the digesters during the pretreatment by CI **(A)**, SI **(B)**, CM **(C)**, SM **(D)**, and DE **(E)**.

Figure 2 exhibits the variation in O_2_ and CO_2_ concentrations in the digesters during the pretreatment with five microbial agents. The trends showed that regardless of the microbial agent used, CO_2_ concentration steadily increased at the beginning of pretreatment along with the consumption of O_2_. CO_2_ and O_2_ concentrations finally reached a stable state within 5 days. Besides, the greater amounts of O_2_ injected into digesters corresponded to higher CO_2_ production. Thus, it could be inferred that all of these agents contained various aerobic or facultative microbes that converted O_2_ into CO_2_ for their growth. More interestingly, at the same oxygen loading, the rates of O_2_ consumption and CO_2_ production in SI, SM, and DE groups were faster than those of CI and CM groups, and the time to exhaust O_2_ was 2 days, even for the highest oxygen loading groups (30 ml/gVS). The results indicated that the aerobic or facultative microbes in SI, SM, and DE groups were more active or abundant, which might possibly contribute to the higher hydrolysis rate.

### Effect of Microbial Pretreatment on Daily Methane Yield

**FIGURE 3:**
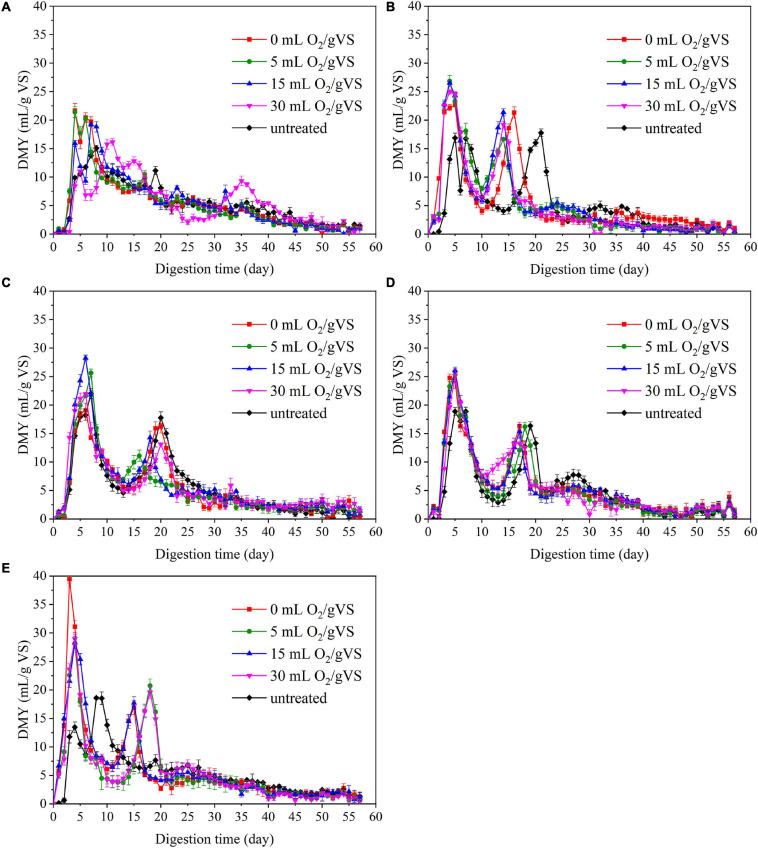
The daily methane yield (DMY) untreated and pretreated PW by CI **(A)**, SI **(B)**, CM **(C)**, SM **(D)**, and DE **(E)**.

The trends in daily methane yield (DMY) of PW pretreated by different microbial agents are shown in Figure 3. The DMY of PW pretreated by CI exhibited an outstanding peak between the 5th and 10th day. Then, there was a swift decrease until another small peak appeared around the 35th day. However, the trends of DMY of PW pretreated by SI, CM, SM, and DE were quite different. There are two peaks observed: one was around the 5th day, and another was between the 10th and 20th days. This phenomenon was related to the accumulation of intermediate products (e.g., VFAs), which has already been reported by Li et al. (2020). The maximum DMY of untreated PW mixed with CI, SI, CM, SM, and DE were 15.1, 16.9, 22.0, 18.9, and 13.5 ml/gVS, respectively. The maximum DMY of PW pretreated by different microbial agents were observed in the groups of CI-0 (21.7 ml/gVS), SI-5 (26.8 ml/gVS), CM-15 (28.2 ml/gVS), SM-15 (26.0 ml/gVS), and DE-15 (39.4 ml/gVS), respectively, with the increase of 43.7, 58.6, 28.2, 37.6, and 191.9% compared with untreated, respectively. The noticeable improvement might be related to diverse microbes that degrade the organic matter to small molecules such as VFAs during the pretreatment.

### Effect of Microbial Pretreatment on Cumulative Methane Yield and Biodegradability

**FIGURE 4:**
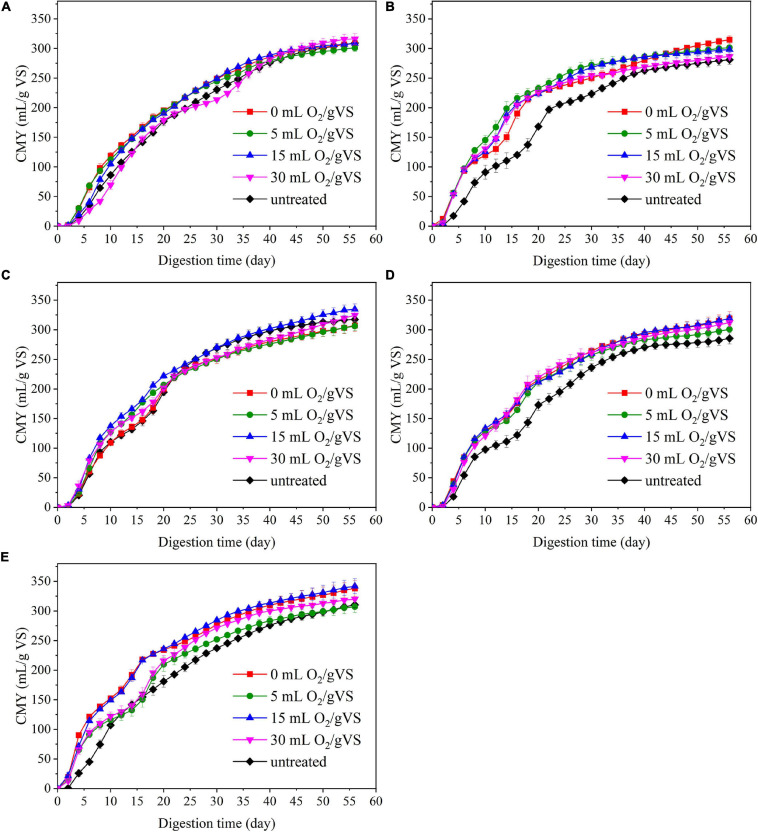
The cumulative methane yield (CMY) of untreated and pretreated PW by CI **(A)**, SI **(B)**, CM **(C)**, SM **(D)**, and DE **(E)**.

Figure 4 and Table 2 present the cumulative methane production of untreated and pretreated PW by microbial agents. Generally, the AD experiment can be divided into two stages by taking the 20th day as the dividing line. During the first 20 days of AD, when DMY remained relatively high, the increase of CMY of all groups was faster than those in the next 40 days. The CMY in the first 20 days (CMY_20_) of CI-UN, SI-UN, CM-UN, SM-UN, and DE-UN were 177.5, 168.1, 194.7, 173.0, and 181.1 ml/gVS, respectively. The enhanced CMY_20_ was achieved after microbial pretreatment. The CMY_20_ of PW in CI-0 was 195.3 ml/gVS, with the highest increase of 10.0% compared with the untreated. The maximum CMY_20_ of PW pretreated by CM was 222.0 ml/gVS in CM-15, with an increase of 14.0% compared with the untreated. However, when the oxygen loading increased to 30 ml/gVS, the CMY_20_ of PW pretreated by CI and CM gradually fell, with an increase of only 0.9 and 3.5%, respectively. Compared with CI and CM, the microbial pretreatments by SI, SM, and DE were more effective in enhancing methane yield at the early stage of AD. The maximum CMY_20_ of PW pretreated by SI, SM, and DE were 232.7 ml/gVS in SI-5, 219.6 ml/gVS in SM-30, and 235.8 ml/gVS in DE-15, with an increase of 38.4, 26.9, and 30.2% compared with untreated. Besides, it is found that changes in oxygen loading during the pretreatment of SI, SM, and DE had a slight influence on the improvement of CMY_20_.

**TABLE 2:** Digestion performance of untreated and pretreated PW.

Group	CMY_20_	CMY_20_ improvement (%)	BD_20_ (%)	EMY	EMY improvement (%)	BD (%)
CI-0	195.3 ± 4.0	10.0	45.1	309.7 ± 3.6	−0.3	71.6
CI-5	194.2 ± 4.6	9.4	44.9	302.0 ± 6.3	−2.8	69.8
CI-15	190.2 ± 3.0	7.1	44.0	309.5 ± 1.4	−0.4	71.5
CI-30	179.0 ± 7.4	0.9	41.4	316.9 ± 8.1	2.0	73.2
CI-UN	177.5 ± 8.1	–	41.0	310.7 ± 3.9	–	71.8
SI-0	224.6 ± 2.7	33.6	51.9	315.6 ± 7.0	12.3	72.9
SI-5	232.7 ± 5.7	38.4	53.8	301.6 ± 3.8	7.3	69.7
SI-15	223.7 ± 5.4	33.0	51.7	298.8 ± 5.3	6.3	69.1
SI-30	226.1 ± 7.5	34.5	52.3	286.9 ± 3.7	2.0	66.3
SI-UN	168.1 ± 2.1	–	38.9	281.2 ± 8.3	–	65.0
CM-0	200.2 ± 7.4	2.8	46.3	307.2 ± 10.2	−3.3	71.0
CM-5	206.6 ± 3.6	6.1	47.8	307.7 ± 7.2	−3.1	71.1
CM-15	222.0 ± 4.5	14.0	51.3	336.0 ± 9.0	5.8	77.7
CM-30	201.5 ± 7.3	3.5	46.6	325.9 ± 10.6	2.6	75.3
CM-UN	194.7 ± 5.7	–	45.0	317.7 ± 5.5	–	73.4
SM-0	215.2 ± 10.1	24.4	49.7	320.6 ± 11.5	12.2	74.1
SM-5	212.1 ± 7.2	22.6	49.0	301.4 ± 6.0	5.5	69.6
SM-15	211.4 ± 2.6	22.2	48.9	320.2 ± 6.9	12.0	74.0
SM-30	219.6 ± 10.7	26.9	50.7	312.7 ± 6.9	9.4	72.3
SM-UN	173.0 ± 9.4	–	40.0	285.7 ± 9.5	–	66.0
DE-0	234.3 ± 0.3	29.3	54.1	339.1 ± 13.8	8.8	78.4
DE-5	209.9 ± 11.9	15.9	48.5	307.6 ± 10.1	−1.3	71.1
DE-15	235.8 ± 1.2	30.2	54.5	343.2 ± 13.3	10.1	79.3
DE-30	216.0 ± 9.0	19.2	49.9	320.6 ± 3.0	2.9	74.1
DE-UN	181.1 ± 9.8	–	41.9	311.6 ± 3.6	–	72.0

*CMY_20_, cumulative methane yield in the first 20 days; BD_20_, BD of the PW in the first 20 days; EMY, experimental methane yield; BD, final BD of the PW; UN, untreated.*

When the AD process came to an end, the EMY of each group could be attained. The highest EMY of pretreated PW was 343.2 ml/gVS in DE-15, with an increase of 10.1% compared with the untreated. The maximum BD of 79.3% was also from this group. Table 3 provides a literature summary regarding the methane production of PW. It is clear that the organic loading in this study was the highest (47.7 gVS/L), followed by 25 gVS/L in the study of Yuan et al. (2014) and 15 gVS/L in that of Li et al. (2020). On the other hand, the methane yield of 343.2 ml/gVS in this study was higher than the estimated methane yield (315.4 ml/gVS) of untreated PW, which was the weighted sum of maximum methane yield of untreated corrugated paper (Krause et al., 2018) and tissue paper (Li et al., 2020) at a ratio of 1 to 1. These findings indicated that with the help of microaerobic pretreatment by DE, the AD of PW might be conducted at a smaller reactor with high organic loading and achieve satisfactory biomethanation efficiency. In addition, the maximum EMY of PW pretreated by other microbial agents were 316.9 ml/gVS in CI-30, 315.6 ml/gVS in SI-0, 336.0 ml/gVS in CM-15, and 320.6 ml/gVS in SM-0, increasing by 2.0, 12.3, 5.8, and 12.2% compared with untreated, respectively. It was noteworthy that the improvement in EMY was not as great compared with that of CMY_20_ for all groups except CI-30, demonstrating that microbial pretreatment tended to accelerate the methane production rate at the early stage of AD. Taken into account the amount or improvement of DMY, CMY_20,_ and EMY, the anaerobic pretreatment or microaerobic pretreatment by SI, SM, and DE were attractive schemes for attaining better methane production performance of PW and deserved deeper investigation in the next sections.

**TABLE 3:** Comparison of highest methane production of various paper wastes from reported literature and this study.

Substrate	Pretreatment method	Organic loading (gVS/L)	Methane production (ml/gVS)	Reference
Waste paper	Microaerobic pretreatment	47.7	343.2	This study
Corrugated paper	None	2.0	272.0	Krause et al., 2018
Paperboard	None	2.0	273.0	Krause et al., 2018
Office paper	None	2.0	375.0	Krause et al., 2018
Toilet paper	None	6.25	348.0	Kim et al., 2019
Office paper	Microbial pretreatment	25.0	221.0	Yuan et al., 2014
Waste paper	Mechanic pretreatment	Not mentioned	253.6	Rodriguez et al., 2017
Corrugated board	None	15.0	243.9	Li et al., 2020
Office paper	None	15.0	284.5	Li et al., 2020
Tissue paper	None	15.0	358.8	Li et al., 2020
Magazine paper	None	10.0	316.4	Li et al., 2020

### VFA Compositions After Microbial Pretreatment by SI, SM, and DE

VFAs represent a series of intermediate products generated from digestible substances and are precursors for methane production. The VFA compositions and concentrations in the anaerobic and microaerobic pretreatment groups by SI, SM, and DE are shown in Table 4. Evidently, the total acid concentration after the microaerobic pretreatment was higher than those of anaerobic pretreatment, indicating that a small amount of oxygen during the pretreatment could help microbes degrade PW into VFAs. At the same oxygen loading level, the pretreated groups by SI possessed the highest total acid concentration in comparison with the SM and DE, implying that SI might harbor more microbes that were efficient in the acidogenesis of PW. The composition of VFAs included various short-chain fatty acids, among which acetic acid took the highest percentage of total acids in all groups. The highest acetic acid concentration was 2846.7 mg/L in SI-0, followed by 2522.8 mg/L in SI-15 and 2456.6 mg/L in DE-15. Since acetic acid could be efficiently converted into methane by acetotrophic methanogens, its production during the pretreatment had positive effects on the AD performance of PW. By comparison, the groups with SM and DE possessed a relatively higher concentration of propionic acid, while n-butyric acid and isovaleric acid were abundant in SI and SM groups. The findings suggested that there existed many microbes in SI, SM, and DE capable of converting the substrates into different VFAs.

**TABLE 4:** VFA composition and concentration of PW after microbial pretreatment of SI, SM, and DE*.

Group	Acetic acid	Propionic acid	Isobutyric acid	n-Butyric acid	Isovaleric acid	Total acid
SI-0	2846.7 ± 181.2	37.7 ± 2.8	54.2 ± 1.0	441.9 ± 9.9	23.8 ± 0.9	3404.3 ± 52.6
SI-15	2522.8 ± 211.6	22.0 ± 6.4	12.4 ± 4.3	1344.4 ± 36.8	4.0 ± 1.3	3905.6 ± 54.1
SM-0	2040.4 ± 129.6	514.9 ± 28.5	59.0 ± 2.1	487.9 ± 20.2	85.1 ± 5.2	3187.3 ± 98.7
SM-15	2175.5 ± 136.3	309.4 ± 16.7	46.9 ± 6.5	1149.4 ± 27.4	43.9 ± 2.6	3725.0 ± 278.6
DE-0	2206.5 ± 146.9	398.0 ± 18.6	41.5 ± 19.1	1.5 ± 0.0	0.0 ± 0.0	2647.5 ± 261.4
DE-15	2456.6 ± 102.5	580.3 ± 24.2	15.1 ± 1.3	0.4 ± 0.0	0.0 ± 0.0	3052.5 ± 68.0

*Unit: mg/L.

### Microbial Community Analysis During Pretreatment and AD

#### Evolution of Microbial Community During Microbial Pretreatment by SI, SM, and DE

**FIGURE 5:**
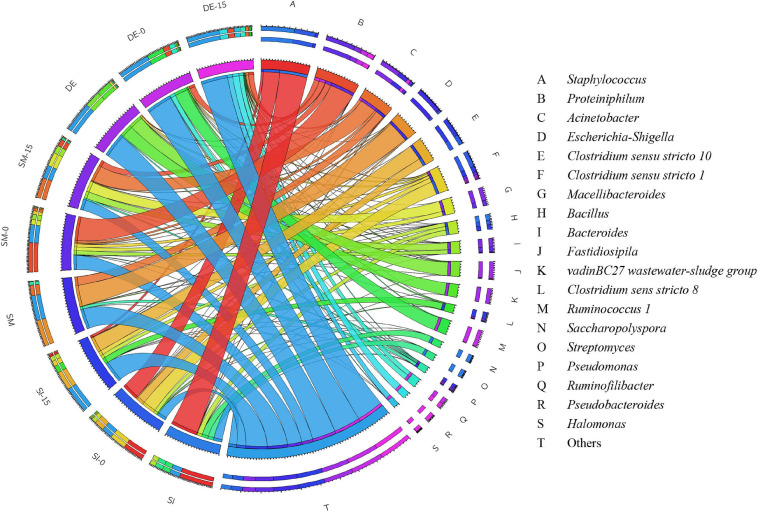
The relative abundance of bacteria in the SI, SM, DE, and pretreated PW at the genus level.

The overview of RA of predominant bacteria in SI, SM, DE, and pretreated PW is provided in Figure 5 and Supplementary Table S1. *Staphylococcus* and *Bacillus* were the predominant genera in SI, whose RAs were 51.42 and 9.07%, respectively. After the anaerobic pretreatment, the RA of *Bacillus* decreased slightly to 6.82%, while that of *Staphylococcus* decreased significantly to 31.52%. Besides, *Clostridium sensu stricto 1* and *Clostridium sensu stricto 10* appeared in SI-0 with the RAs of 25.10 and 15.27%, respectively. Members of *Bacillus* are aerobes or facultative anaerobes that produce cellulase, hemicellulase, ligninase, amylase, protease, and pectinase (Dastager et al., 2015; Yang et al., 2017; Xu et al., 2018). Therefore, this genus might result in the rapid consumption of oxygen during the pretreatment (as shown in Figure 2B) and induce cellulose and lignin degradation. *Staphylococcus* was regarded as a facultative anaerobic organism that could convert various sugars to organic acids (Supré et al., 2010; De Bel et al., 2013) and was possibly the main contributor to anaerobic pretreatment by SI. *Clostridium sensu stricto 1* and *Clostridium sensu stricto 10* accounted for a large proportion in SI-0. *Clostridium* included a variety of bacteria that specialized in utilizing multiple sugars as carbon and energy sources to generate methanogenic precursors such as acetic acid, butyric acid, H_2_, and CO_2_ (Lanjekar et al., 2015; Amin et al., 2021). Compared with SI-UN, the higher RA of *Clostridium* in SI-0 might contribute to the acceleration of methane production at the early stage, as shown in Figure 4B. The SM and DE also contained various hydrolysis and acidogenesis bacteria. The RAs of *Bacteroides* and *Macellibacteroides* in SM were 0.63 and 0.15%, respectively, but increased by dozens and hundreds of times in SM-15, respectively. Many bacteria in these genera could convert cellulose into various VFAs (Jabari et al., 2012; Sakamoto and Ohkuma, 2013; Hatamoto et al., 2014). The RAs of *Proteiniphilum* in SM and DE were 0.08 and 1.24%, respectively, but significantly increased after microbial pretreatment. It was reported that *Proteiniphilum* functioned by converting the proteins and pyruvate into acetate and propionate (Chen and Dong, 2005). Noticeably, *Acinetobacter* was abundant in SM (15.87%) and SM-15 (31.16%). Most bacteria from this genus were aerobic, and some were associated with lignin degradation (Radolfova-Krizova et al., 2016; Yang et al., 2017), contributing to the rapid oxygen consumption (shown in Figure 2D) and higher methane production of pretreated PW.

#### Changes of Microbial Community After AD

Table 5 provides information regarding the species diversity and richness of bacteria and archaea in selected groups after AD, including sequence number, OTU number, ACE, Chao1, Shannon, and Simpson indexes. After the pretreatment by SI, the highest sequence number, OTU number, Shannon, ACE, and Chao1 indexes of bacteria in bacteria were achieved in SI-15, followed by SI-UN and SI-0. However, the observed outcomes in the groups of SM were quite different. These indexes of bacteria in SM-UN were the highest, followed by SM-0 and SM-15. A possible explanation for this result might be that the vulnerable bacteria in SM gradually disappeared due to the changes in the environment in the digester. The Shannon, ACE, and Chao1 indexes of bacteria in DE-0 and DE-15 showed a minor difference with those of DE-UN, indicating that many bacteria in the digestate were not susceptible to interference during the pretreatment and AD. The variations of species diversity and richness of archaea were quite different from those of bacteria. For SI and DE, the sequence number of archaea in the groups of anaerobic pretreatments higher than the untreated group and microaerobic pretreatment group. Besides, the sequence number and OTU number of archaea in SM-0 was higher than that in SM-15. It could be inferred that the anaerobic pretreatment was beneficial for methanogens to keep their activity during the AD.

**TABLE 5:** Ecological indexes of bacteria and archaea in selected groups after AD.

	Group	Sequence number	OTU number	Shannon	ACE	Chao1	Coverage	Simpson
Bacteria	SI-0	43202	1144	4.25	2007.24	1591.66	0.99	0.05
	SI-15	73884	1522	4.59	2571.82	2182.26	0.99	0.03
	SI-UN	64742	1354	4.59	2425.33	2006.00	0.99	0.03
	SM-0	64964	1658	4.75	2444.58	2424.27	0.99	0.02
	SM-15	61462	1392	4.54	2440.20	2338.35	0.99	0.03
	SM-UN	75071	1742	5.00	2906.55	2509.54	0.99	0.02
	DE-0	57959	1783	4.66	2568.92	2424.17	0.99	0.03
	DE-15	52705	1558	4.75	2894.59	2358.81	0.99	0.03
	DE-UN	56081	1446	4.65	2483.26	2029.88	0.99	0.02
Archaea	SI-0	64353	471	1.93	1250.39	805.60	1.00	0.27
	SI-15	57131	400	1.69	906.49	680.73	1.00	0.32
	SI-UN	58345	424	1.81	771.91	732.37	1.00	0.27
	SM-0	64300	548	1.54	1242.33	970.27	1.00	0.46
	SM-15	47132	369	1.80	1323.66	872.52	1.00	0.32
	SM-UN	65814	473	2.24	1298.76	881.37	1.00	0.16
	DE-0	69012	520	1.21	1346.49	956.11	1.00	0.59
	DE-15	60695	457	2.15	961.04	707.87	1.00	0.20
	DE-UN	61481	373	1.73	1427.72	803.18	1.00	0.29

**FIGURE 6:**
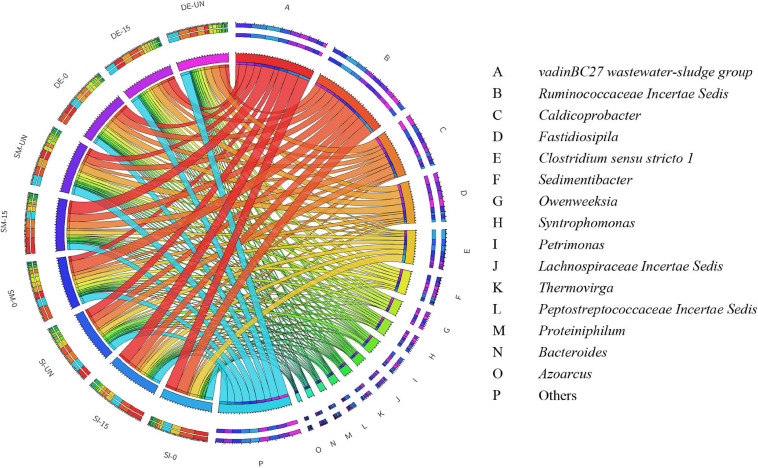
The relative abundance of bacteria in the selected digestates after AD at the genus level.

Figure 6 and Supplementary Table S2 exhibit the overview of abundant bacteria in selected groups after AD. Evidently, the microbial community in each digester was significantly changed. Some remarkable bacteria for pretreatment, like *Proteiniphilum*, *Bacillus*, and *Staphylococcus*, were nearly absent, while *VadinBC27* and *Ruminococcaceae Incertae Sedis* appeared as the predominant bacteria in all digesters. From previous studies, *VadinBC27* was recognized as degrading amino acids in syntrophic association with hydrogenotrophic methanogens (Li et al., 2015). *Ruminococcaceae Incertae Sedis* was previously identified as a functional bacterium associated with cellulose and hemicellulose degradation (Ecem Öner et al., 2018). Therefore, the microbial community containing these bacteria enabled the enhancement of methane production. *Clostridium sensu stricto 1* was another primary bacterium in the digesters with SI. The RA of *Clostridium sensu stricto 1* in SI-0 was 16.23%, with an increase of 58.6% compared with SI-UN. Noticeably, *Clostridium sensu stricto 1* took up a large part after the pretreatment and possessed high abundance after AD. These results suggested that it was adaptive to the environment in digesters and might play critical roles in both microbial pretreatment and AD. Besides, *Fastidiosipila* and *Caldicoprobacter* were also observed in the digesters with SM and DE after AD. The former was able to produce acetate and butyrate from carbohydrates and protein (Falsen et al., 2005), and the latter could metabolize cellulose and hemicellulose into oligosaccharides or monosaccharides (Zhou et al., 2018). Aside from the predominant bacteria mentioned above, others detected in these digestates include *Sedimentibacter*, *Syntrophomonas*, *Owenweeksia*, and *Petrimonas*. The RAs of *Sedimentibacter* in SI-0 and SI-15 were 3.70 and 3.89%, respectively, with an increase of 30.0 and 36.9% compared with the untreated (2.84%). It has been reported that *Sedimentibacter* was identified as a strict anaerobe that could degrade amino acids into ethanol and organic acids (Lechner, 2015; Imachi et al., 2016). *Syntrophomonas* facilitates the conversion of butyric acid into acetate, propionate, and H_2_ under methanogenic conditions (Sekiguchi, 2015), and its RA ranged between 1.54 and 5.41% in the digestates we collected. The RA of *Petrimonas* experienced a minor difference in the groups of SM (1.67–2.47%) and DE (3.69–4.23%). This genus was an important participant during AD as it could ferment many sugars and organic acids into methane production precursors like acetate, H_2_, and CO_2_ (Grabowski et al., 2005).

**FIGURE 7:**
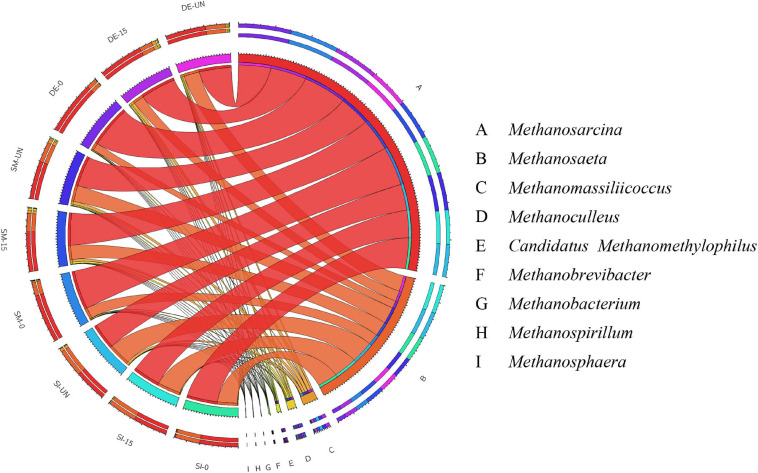
The relative abundance of archaea in the selected digestates after AD at the genus level.

The community structures of archaea in selected groups after AD are presented in Figure 7 and Supplementary Table S3. Obviously, *Methanosarcina*, and *Methanosaeta* were the main methanogens in all groups regardless of microbial agents and oxygen loading for pretreatment. By comparing these two genera in the same microbial agent groups, it could be found that the groups with anaerobic pretreatment possessed the highest RA of *Methanosarcina*, followed by those with microaerobic pretreatment and without pretreatment, while the RA of *Methanosaeta* showed opposite trends. For example, the RAs of *Methanosarcina* and *Methanosaeta* in SM-UN were 59.13 and 31.60%, respectively. *Methanosarcina* significantly increased to 62.51 and 75.53% in SM-15 and SM-0, respectively, while the *Methanosaeta* decreased to 28.27 and 21.33%. *Methanosarcina* is known as the only methanogen compatible with the acetoclastic and hydrogenotrophic methanogenesis routes, while *Methanosaeta* has the ability to follow the acetoclastic pathway only (Zakaria and Dhar, 2019). The shift between *Methanosarcina* and *Methanosaeta* might probably result from the changes of precursors produced by the pretreatment with different microbial agents and oxygen loading and consequently bring about better performance of methane production of PW.

## Conclusion

This study was designed to investigate the methane production performance of PW subjected to anaerobic and microaerobic pretreatments by five microbial agents, namely, CI, SI, CM, SM, and DE. Results showed the diverse efficacy of these microbial agents and oxygen loadings on the methane production performance of PW. The EMY of pretreated PW was enhanced by the pretreatments of SI, SM, and DE under an optimal oxygen loading. The microbial community analysis revealed that microbial agents provided functional bacteria for enhancing the hydrolytic and acidogenic process, which exhibited a different response to the oxygen loading during the anaerobic and microaerobic pretreatment. Besides, the anaerobic and microaerobic pretreatment had a profound effect on the microbial community in AD digesters, leading to the enhanced methane production performance. The work not only provided a promising technique to make full use of PW but also gave a reference for further studies on biodegradation mechanisms of lignocellulosic biowastes during the microbial pretreatment at various oxygen concentrations and AD processes.

## Data Availability Statement

The raw data supporting the conclusions of this article will be made available by the authors, without undue reservation.

## Author Contributions

CS and WL conducted the experiment and wrote the original draft. FC performed the statistical analysis. GL provided the resources for this experiment. CC contributed to the conception and supervision of this study. All authors contributed to manuscript revision, read, and approved the submitted version.

## Conflict of Interest

The authors declare that the research was conducted in the absence of any commercial or financial relationships that could be construed as a potential conflict of interest.

## Footnotes

1 http://circos.ca/intro/tabular_visualization/
