# Supplementary material for: Anaerobic and Microaerobic Pretreatment for Improving Methane Production From Paper Waste in Anaerobic Digestion
Source: Front Microbiol. 2021 Jul 6;12:688290. doi: 10.3389/fmicb.2021.688290 (PMC8290346; doi:10.3389/fmicb.2021.688290)
Supplement: Supplementary file 1 [file Data_Sheet_1.docx]

Supplementary Material

# Supplementary Tables

**Table S1.** The relative abundance of bacteria in the SI, SM, DE, and pretreated PW at the genus level.

| ID | SI | SI-0 | SI-15 | SM | SM-0 | SM-15 | DE | DE-0 | DE-15 |
| --- | --- | --- | --- | --- | --- | --- | --- | --- | --- |
| *Staphylococcus* | 51.42% | 31.52% | 3.83% | 0.00% | 0.00% | 0.00% | 0.00% | 0.00% | 0.00% |
| *Proteiniphilum* | 0.01% | 0.09% | 0.01% | 0.08% | 44.97% | 10.83% | 1.24% | 10.71% | 10.14% |
| *Acinetobacter* | 0.16% | 0.02% | 1.16% | 15.87% | 3.96% | 31.16% | 0.18% | 0.17% | 4.88% |
| *Escherichia-Shigella* | 0.03% | 2.47% | 0.01% | 39.87% | 2.52% | 0.55% | 0.00% | 0.00% | 0.03% |
| *Clostridium sensu stricto 10* | 0.00% | 15.27% | 29.22% | 0.00% | 0.01% | 0.00% | 0.00% | 0.00% | 0.00% |
| *Clostridium sensu stricto 1* | 0.00% | 25.10% | 7.69% | 0.00% | 5.03% | 0.98% | 3.01% | 2.02% | 1.39% |
| *Macellibacteroides* | 0.00% | 0.00% | 0.00% | 0.15% | 7.69% | 20.16% | 0.00% | 0.13% | 0.00% |
| *Bacillus* | 9.07% | 6.82% | 6.65% | 0.09% | 0.01% | 0.05% | 0.07% | 0.00% | 0.12% |
| *Bacteroides* | 0.00% | 0.01% | 0.00% | 0.63% | 7.50% | 12.92% | 0.91% | 0.22% | 0.12% |
| *Fastidiosipila* | 0.01% | 0.00% | 0.00% | 0.00% | 0.04% | 0.00% | 25.07% | 1.11% | 0.98% |
| *vadinBC27 wastewater-sludge group* | 0.01% | 0.00% | 0.00% | 0.00% | 0.00% | 0.00% | 22.86% | 0.00% | 0.00% |
| *Clostridium sens stricto 8* | 0.00% | 1.34% | 11.22% | 0.00% | 0.00% | 0.77% | 0.00% | 1.34% | 0.37% |
| *Ruminococcus 1* | 0.00% | 0.00% | 0.00% | 0.00% | 0.00% | 0.00% | 0.00% | 25.24% | 1.15% |
| *Saccharopolyspora* | 11.80% | 0.24% | 0.21% | 0.00% | 0.00% | 0.00% | 0.00% | 0.00% | 0.00% |
| *Streptomyces* | 11.66% | 0.15% | 0.08% | 0.00% | 0.00% | 0.00% | 0.00% | 0.00% | 0.00% |
| *Pseudomonas* | 0.15% | 0.00% | 0.02% | 5.50% | 0.82% | 2.33% | 1.72% | 0.17% | 0.85% |
| *Ruminofilibacter* | 0.00% | 0.00% | 0.00% | 0.00% | 0.00% | 0.00% | 0.00% | 9.11% | 7.82% |
| *Pseudobacteroides* | 0.00% | 0.00% | 0.00% | 0.00% | 0.00% | 0.00% | 0.00% | 0.35% | 12.52% |
| *Halomonas* | 0.00% | 0.00% | 0.00% | 0.01% | 0.01% | 0.00% | 0.00% | 0.07% | 9.48% |
| Others | 15.68% | 16.98% | 39.89% | 37.80% | 27.43% | 20.24% | 44.94% | 49.37% | 50.16% |

**Table S2.** The relative abundance of bacteria in the selected digestates after AD at the genus level.

| ID | SI-0 | SI-15 | SI-UN | SM-0 | SM-15 | SM-UN | DE-0 | DE-15 | DE-UN |
| --- | --- | --- | --- | --- | --- | --- | --- | --- | --- |
| *vadinBC27 wastewater-sludge group* | 23.12% | 25.05% | 17.32% | 10.15% | 25.95% | 14.20% | 15.57% | 15.34% | 6.74% |
| *Ruminococcaceae Incertae Sedis* | 22.47% | 17.36% | 13.21% | 23.74% | 15.89% | 14.57% | 16.52% | 13.92% | 13.49% |
| *Caldicoprobacter* | 2.88% | 7.48% | 15.89% | 6.22% | 15.49% | 6.49% | 10.53% | 7.67% | 17.27% |
| *Fastidiosipila* | 3.60% | 4.87% | 3.70% | 9.11% | 4.07% | 14.38% | 12.93% | 15.01% | 12.35% |
| *Clostridium sensu stricto 1* | 16.23% | 10.01% | 10.23% | 8.33% | 7.30% | 3.09% | 3.26% | 5.31% | 1.70% |
| *Sedimentibacter* | 3.70% | 3.89% | 2.84% | 7.91% | 3.15% | 6.79% | 5.97% | 5.79% | 6.18% |
| *Owenweeksia* | 0.19% | 0.01% | 0.03% | 6.26% | 0.42% | 5.70% | 6.77% | 4.66% | 6.41% |
| *Syntrophomonas* | 1.54% | 3.35% | 2.25% | 3.73% | 2.38% | 5.41% | 3.51% | 3.92% | 4.13% |
| *Petrimonas* | 2.19% | 2.48% | 6.78% | 1.67% | 1.98% | 2.47% | 4.23% | 3.90% | 3.69% |
| *Lachnospiraceae Incertae Sedis* | 1.29% | 1.06% | 2.64% | 1.73% | 3.28% | 2.35% | 2.42% | 2.43% | 2.72% |
| *Thermovirga* | 1.69% | 2.48% | 1.88% | 1.57% | 1.65% | 2.07% | 2.24% | 2.05% | 2.27% |
| *Peptostreptococcaceae Incertae Sedis* | 2.61% | 1.89% | 3.61% | 1.29% | 1.73% | 1.05% | 0.89% | 1.20% | 0.84% |
| *Proteiniphilum* | 1.65% | 1.84% | 1.50% | 1.23% | 0.91% | 2.04% | 0.27% | 1.51% | 1.26% |
| *Bacteroides* | 0.53% | 1.72% | 1.41% | 1.53% | 1.95% | 1.63% | 1.04% | 1.00% | 1.25% |
| *Azoarcus* | 0.79% | 1.31% | 0.77% | 0.80% | 0.87% | 1.02% | 0.39% | 0.58% | 0.44% |
| Others | 15.51% | 15.21% | 15.94% | 14.72% | 12.99% | 16.72% | 13.45% | 15.73% | 19.29% |

**Table S3.** The relative abundance of archaea in the selected digestates after AD at the genus level.

| ID | SI-0 | SI-15 | SI-UN | SM-0 | SM-15 | SM-UN | DE-0 | DE-15 | DE-UN |
| --- | --- | --- | --- | --- | --- | --- | --- | --- | --- |
| *Methanosarcina* | 59.66% | 53.06% | 50.84% | 75.53% | 62.51% | 59.13% | 82.90% | 69.28% | 62.56% |
| *Methanosaeta* | 38.26% | 44.89% | 43.31% | 21.33% | 28.27% | 31.60% | 14.29% | 20.27% | 30.61% |
| *Methanomassiliicoccus* | 0.92% | 0.51% | 4.65% | 0.67% | 4.50% | 5.67% | 0.63% | 6.25% | 3.20% |
| *Methanoculleus* | 0.76% | 1.22% | 0.69% | 1.42% | 3.55% | 1.95% | 1.51% | 2.44% | 1.63% |
| *Candidatus_Methanomethylophilus* | 0.22% | 0.17% | 0.39% | 0.20% | 0.95% | 1.33% | 0.27% | 1.25% | 1.57% |
| *Methanobrevibacter* | 0.04% | 0.05% | 0.02% | 0.73% | 0.13% | 0.22% | 0.24% | 0.38% | 0.38% |
| *Methanobacterium* | 0.04% | 0.02% | 0.06% | 0.05% | 0.04% | 0.02% | 0.11% | 0.06% | 0.01% |
| *Methanospirillum* | 0.10% | 0.07% | 0.04% | 0.02% | 0.02% | 0.05% | 0.04% | 0.03% | 0.02% |
| *Methanosphaera* | 0.01% | 0.01% | 0.01% | 0.05% | 0.02% | 0.03% | 0.01% | 0.03% | 0.02% |
